# African Swine Fever: Epidemiology, the Design of New Diagnostic Methods, and Vaccine Development

**DOI:** 10.3390/pathogens12081042

**Published:** 2023-08-15

**Authors:** Marta Martínez-Avilés

**Affiliations:** Infectious Diseases and Global Health Department, Centro de Investigación en Sanidad Animal (CISA), Instituto Nacional de Investigación y Tecnología Agraria y Alimentaria-Consejo Superior de Investigaciones Científicas (INIA-CSIC), 28130 Madrid, Spain; martinez.marta@inia.csic.es

African swine fever (ASF) is a pandemic viral disease that poses a major threat to the health of wild and domestic pigs, national economies, and subsistence livelihoods around the world.

Before 2007 (when ASF appeared in Georgia, Europe), there were fewer than five outbreaks of ASF reported per year (all in Africa). Between 2007 and 2014, cases increased to an average of 77 per year. The disease mainly affected domestic pigs (>170,000) and the ASF virus (ASFV) spread from Georgia to Armenia, Russia, and Belarus. From 2014 to 2018, the ASFV spread through the Baltic region, resulting in an annual average of nearly 300 domestic pig outbreaks (230,000 pigs affected) and over 2000 wild boar cases. In 2018, ASF appeared in China, and the virus rapidly spread in subsequent years throughout the Asian continent, with only a few countries remaining free. In the period 2018–2022, the annual average of affected domestic pigs increased to 1800 and that of affected wild boar increased to 4500. The worst year for domestic pigs was 2019, with 8.5 million pigs affected in Asian regions alone. For wild boar, the worst year was 2020, with 5795 cases and 16,715 wild pigs affected. The virus reached Papua New Guinea in Oceania in 2020 and the Caribbean (Dominican Republic and Haiti) by 2021, resulting in ASF outbreaks on all five continents.

There are three main epidemiological cycles involved in ASF transmission: domestic pigs, wild pigs, and ticks. All three are difficult to control. So far, in the current pandemic, ticks have not been found to play an epidemiological role. However, given the occurrence of ASF in countries with high biodiversity and favourable conditions for tick involvement, this possibility cannot be ruled out in the near future. In the current pandemic, only Belgium (where only wild boar were affected) has been able to eradicate ASF. The insidious nature of the ASFV and its ability to survive in the environment and in frozen and chilled meat enable a slower transmission than other contagious diseases. The involvement of backyard or subsistence pig farming further complicates control efforts. Other factors, such as social factors and an underestimation of risk, promote transmission and perpetuation. The difficulties in controlling ASF in wild boar, and its worldwide distribution have led to increased research endeavours centred around ASF vaccines, which were previously not available to control the disease.

This Special Issue aims to focus on the updated aspects of ASF epidemiology, the design of new diagnostic methods, and vaccine and vaccination developments that help to control and ultimately eradicate the disease in different scenarios.

The collection includes a review by Penrith et al. on pig-adapted ASFV strains inside and outside Africa [1]. ASF originated in a sylvatic cycle in Africa, and of the 24 identified ASFV genotypes, two (genotypes I and II) have spread beyond Africa. Today, the pandemic is caused by ASFV genotype II, but genotypes VIII, IX, and X are also extended within the domestic pig population in Africa. The distribution, history, and characteristics of these pig-adapted genotypes are reviewed in this manuscript. De la Torre et al. explored the risk factors and risk perception of domestic pig and wild boar experts in Europe [2], where wild boar movements and pork and pork product movements were identified as high-risk factors for the introduction and spread of the ASFV. Muñoz-Perez et al. computed a stochastic risk assessment model to assess the risk of ASF introduction into Spain through the legal import of pigs [3]. Although the risk of introduction was found to be low, the consequences could be catastrophic given that Spain is one of the world’s leading pig-producing countries.

In relation to new diagnostic methods, important aspects have been covered. IIya et al. developed a rapid diagnostic method for the detection of ASFV genotypes I and II [4], which is especially relevant due to the recent detection of attenuated ASFV genotype I in illegal vaccines used in China. The method showed a sensitivity rate of 96.8% and a specificity rate of 100%, with 100% agreement to a reference commercial real-time qPCR. Onyilagha et al. evaluated the ability of a commercial lateral flow assay (LFA) to detect ASFV strains in clinical samples from pigs infected with highly virulent ASFV strains [5]. While the LFA was specific, its sensitivity was lower than that of laboratory-based real-time PCR assays. However, the study suggested that the LFA could be a useful herd-level, field-deployable, and easy-to-use diagnostic tool, particularly in hard-to-reach remote areas with limited access to central laboratories during outbreaks. Lastly, Mazloum et al. identified three single locus molecular targets for ASFV phylogenetic practices in wild boar [6], based on a comparison of full genome sequences from samples taken in 2019 from eastern and western regions of Russia.

In relation to vaccine development, Deutschmann et al. experimentally tested the vaccine candidate “ASFV-G-∆MGF” in domestic pigs and wild boar [7]. Their study showed that all vaccine responders were fully protected against challenge infection, although a lower efficiency was observed after oral administration compared to intramuscular administration. 

In conclusion, this Special Issue has fulfilled its objectives, although the global control and prevention of ASF continue to be a high priority around the world and still need further research developments.
